# Paternal high‐fat diet enhances offspring whole‐body insulin sensitivity and skeletal muscle insulin signaling early in life

**DOI:** 10.14814/phy2.13583

**Published:** 2018-02-27

**Authors:** Leslie A. Consitt, Gunjan Saxena, Yuriy Slyvka, Brian C. Clark, Max Friedlander, Yizhu Zhang, Felicia V. Nowak

**Affiliations:** ^1^ Department of Biomedical Sciences Heritage College of Osteopathic Medicine Ohio University Athens Ohio USA; ^2^ Diabetes Institute Ohio University Athens Ohio USA; ^3^ Ohio Musculoskeletal and Neurological Institute Ohio University Athens Ohio USA

**Keywords:** Akt, AS160, diet, father, insulin sensitivity, muscle

## Abstract

Evidence suggests that paternal diet can predispose offspring to metabolic dysfunction. Despite this knowledge, little is known regarding the effects of paternal high‐fat feeding on offspring insulin sensitivity. The purpose of this study was to investigate for the first time the effects of paternal high‐fat feeding on whole‐body and skeletal muscle insulin action in young and adult offspring. At 4 weeks of age, founder C57BL6/N males (F0) were fed a high‐fat diet or control diet for 12 weeks and then bred with females on a control diet. Offspring (F1) were euthanized at 6 weeks, 6 months, or 12 months and insulin‐stimulated insulin signaling was measured ex vivo in isolated soleus muscle. At 6 weeks of age, paternal high fat offspring (HFO) had enhanced whole‐body insulin sensitivity (35%, *P *<* *0.05), as well as, increased insulin‐stimulated skeletal muscle phosphorylation of Akt threonine 308 (70%, *P *<* *0.05) and AS160 threonine 642 (80%, *P *<* *0.05) compared to paternal control fed offspring (CFO), despite both offspring groups consuming standard chow. At 6 months of age, HFO had increased percent body fat compared to CFO (74%, *P *<* *0.005) and whole‐body and skeletal muscle insulin signaling normalized to CFO. Body fat was inversely related with insulin signaling in HFO, but not CFO. These findings suggest that paternal high‐fat feeding contributes to enhanced whole‐body and skeletal muscle insulin sensitivity in HFO early in life; however, these benefits are lost by early adulthood, potentially due to premature increases in body fat.

## Introduction

Rates of obesity and type 2 diabetes have reached epidemic proportions over the past decade with researchers continuing efforts to elucidate the factors responsible for weight gain and insulin resistance. An alarming statistic reveals that in the United States and Canada, the prevalence of obesity is growing most rapidly in young adults (20–39‐year olds) (Shields et al. 2011; WHO 2016), which will have adverse social and economic consequences for the future. Given the current and future health concerns, the need to identify mechanism(s) responsible for predisposing an individual to obesity and insulin resistance is critical (Li et al. 2009; Cooper et al. 2010; Freeman et al. 2012).

Obesity and insulin resistance have traditionally been linked to environmental and/or genetic inheritance factors (Stunkard et al. 1986; Bouchard et al. 1988, 1990; Drewnowski 2004); however, recent evidence suggests that diet‐induced obesity and/or altered glucose homeostasis in the mother or father can predispose or “program” offspring for metabolic dysfunction, independent of genetic inheritance (Fullston et al. 2013; Slyvka et al. 2015; Nicholas et al. 2016; Shi et al. 2017). Early studies focused on the physiological effects of maternal obesity, reporting that offspring had impairments in body composition, glucose tolerance, insulin sensitivity and lipid metabolism (Warner and Ozanne 2010; Dunn and Bale 2011; Fullston et al. 2013; Huypens et al. 2016; Nicholas et al. 2016). More recently, the role of paternal “programming” has gained interest with studies reporting that male and female offspring of obese fathers had dramatically increased risk for the development of obesity, regardless of the mother's weight (Cooper et al. 2010; Freeman et al. 2012). While epidemiological studies highlight important associations between parent and child, experimental rodent studies have allowed the control of environmental conditions. Of particular interest, it was recently reported that the offspring of paternal high‐fat diet‐fed rodents developed increased adiposity as early as 8 weeks of age (Fullston et al. 2013; Lecomte et al. 2017) and two studies reported the onset of insulin resistance at 16–24 weeks of age (Fullston et al. 2013; Masuyama et al. 2016) despite the consumption of control chow in the offspring, suggesting the father's diet prior to conception could predispose offspring to the development of type 2 diabetes. Based on previously published time points, it remains unclear if insulin resistance occurs prior to adulthood and what cellular mechanism(s) may contribute to the insulin resistance in these mice.

Skeletal muscle plays a prominent role in determining whole‐body glucose regulation and is considered the primary target for insulin‐stimulated glucose uptake (DeFronzo 1981). Insulin binds to its receptor, initiating a signaling cascade resulting in the translocation of the glucose transporter 4 (GLUT4) to the plasma membrane to allow glucose uptake into the muscle (Thorell et al. 1999). The Akt substrate of 160 kDa (AS160, also known as TBC1D4) is currently recognized as the most distal signaling step necessary for insulin‐stimulated glucose uptake. In response to insulin, AS160 is phosphorylated on a number of Akt consensus sequences, allowing the translocation of GLUT4 to the plasma membrane (Sano et al. 2003; Cartee and Wojtaszewski 2007; Sakamoto and Holman 2008). We have previously reported that insulin‐stimulated whole‐body insulin sensitivity is positively associated with skeletal muscle AS160 phosphorylation on serine 588 and threonine 642 sites (Consitt et al. 2013). Our group (Consitt et al. 2013) and others (Middelbeek et al. 2013) have also highlighted the necessity to measure site‐specific phosphorylation of AS160 to obtain a better understanding of the impairments in insulin action. Maternal overnutrition has been linked to impairments in offspring skeletal muscle metabolism (Latouche et al. 2014) and recently it was determined that maternal high‐fat feeding elicited impaired skeletal muscle insulin signaling in offspring (Fante et al. 2016). Unfortunately, it remains unknown if paternal high‐fat feeding metabolically programs offspring skeletal muscle.

To our knowledge, the effects of paternal high‐fat diet on offspring skeletal muscle insulin signaling have not been studied. In addition, while previous experimental research indicates that metabolic programming can occur in adulthood, it remains unclear if paternal programming can induce whole‐body and skeletal muscle insulin resistance at a young age. Therefore, the purpose of the current study was to investigate for the first time the effects of paternal high‐fat diet feeding on skeletal muscle insulin signaling in young to mature adult offspring.

## Materials and Methods

### Animals and breeding

Pathogen‐free C57BL6/N mice were obtained from Harlan Sprague Dawley Inc. (Indianapolis, IN). Starting at 4 weeks of age, females were fed a control diet (D12450B, 10% fat, 70% carbohydrate, 20% protein, Research Diets, New Brunswick, NJ) and founder males (F0) were fed either a control (D12450B) or high‐fat diet (D12451, 45% fat, 35% carbohydrate, 20% protein, Research Diets, New Brunswick, NJ) for 12 weeks. Mating pairs were assigned to produce two groups of F1 offspring: ♂ control diet/♀ control diet (control diet father offspring, CFO) and ♂ high‐fat diet/♀ control diet (high‐fat diet father offspring, HFO). Only males fed a high‐fat diet that fit the criteria for obesity (body weight at least 20% greater than age‐ and sex‐matched animals on control diet) were mated. Mice were used only once for mating. Offspring from litters with five to seven pups were used for experiments to control for prenatal and postnatal nutrition. There were six successful litters for CFO and four successful litters for HFO that were used for experiments described below. Offspring from each litter were randomized to three different age groups (6 weeks, 6 months, and 12 months). Females were maintained on the control diet throughout gestation and lactation and all offspring were fed a standard chow diet (Prolab RMH 3000) until euthanasia at either 6 weeks, 6 months, or 12 months of age. All procedures performed with the mice were approved by the Institutional Animal Care and Use Committee at Ohio University, which is AALAC approved, and conducted in accordance with all standards set forth by federal, state, and local authorities.

### Insulin sensitivity test (IST) and body composition

Insulin sensitivity tests (IST) were performed on six litters of CFO and four litters of HFO at 5–6 weeks (CFO: six females, six males; HFO: four females, four males), 6 months (CFO: four females, four males; HFO: four females, four males), or 12 months (CFO: three females, three males; HFO: three females, three males) of age in offspring. After a 6 h fast, the IST was performed by intraperitoneal injection of insulin (Humilin R; Lilly) at a dose of 1 U/kg body weight. Tail blood glucose concentrations were measured using a One Touch Glucometer at 0, 15, 30, 60, and 120 min after injection and used to calculate glucose area under the curve (AUC). Body composition was completed on all mice that were euthanized for skeletal muscle measurements (see Skeletal muscle incubations for sample size breakdown). During the week prior to planned euthanasia, each mouse had percent body fat and percent lean mass measured by nuclear magnetic resonance spectroscopy (NMR) using a minispec (Bruker Optics, The Woodlands, TX).

### Skeletal muscle incubations

Skeletal muscle insulin signaling was performed ex vivo in the soleus muscle of offspring on six litters of CFO and four litters of HFO at 6 weeks (CFO: three females, five males; HFO: two females, four males), 6 months (CFO: three females, three males; HFO: three females, three males), and 12 months (CFO: three females, three males; HFO: three females, three males). Mice were anesthetized with Avertin (250 mg/kg mouse) via intraperitoneal injection and then euthanized by cardiac puncture. Soleus muscle were dissected from each mouse and immediately incubated for 20 min in flasks containing 2 mmol/L of Krebs–Henseleit bicarbonate buffer (KHB) supplemented with 2 mmol/L sodium pyruvate and 6 mmol/L mannitol and placed in a shaking incubator maintained at 35°C gassed with 95% O_2_–5% CO_2_ as explained previously (Gupte et al. 2008). Following initial incubation, one soleus from each mouse was placed in 2 mL of fresh KHB supplemented buffer (basal buffer) and the other soleus was placed in basal buffer plus 100 nmol/L insulin for 20 min (insulin). At the end of incubation, muscle was immediately frozen in liquid nitrogen for later measurement of insulin signaling proteins by western blot procedures.

### Western blot procedure

Soleus muscle was homogenized and protein content was determined as previously described (Consitt et al. 2013, 2016). Muscle lysate (20 *μ*g cellular protein) was separated by SDS‐PAGE, electrotransferred onto polyvinylidene difluoride membranes (Millipore, Billerica, MA), and probed overnight with Cell Signaling (Beverly, MA) antibodies for Akt (Thr308), AS160 (Ser588), AS160 (Thr642), AS160 (Ser318), and AS160 total, or Santa Cruz Biotechnology (Santa Cruz, CA) antibodies for Akt (Ser473), Akt1/2/3 total, and actin. Samples were normalized to a control sample that was resolved on each gel and phosphorylation levels were normalized to their corresponding total protein that was probed after membranes were stripped of the phosphorylation‐specific antibodies, as previously reported (Consitt et al. 2016).

### Statistics

Analyses were performed using SPSS version 21.0 software (SPSS Inc., Chicago, IL). ANOVA procedures were used to examine the interactive effects of paternal group (CFO and HFO), offspring age (6 weeks, 6 months, and 12 months), and offspring biological sex (male, female) on body composition and insulin sensitivity. For skeletal muscle insulin signaling data, a repeated measures ANOVA was used with condition (basal and insulin stimulated), age, and paternal group added to the model. Biological sex was initially added to each model for insulin signaling‐dependent variables; however, because biological sex exerted nonsignificant and minimal effects on the analyses (e.g., eta‐squared effect sizes on the order of 0.02–0.08), data were collapsed across biological sex. Significant interactions were further analyzed using unpaired (paternal group, age group) *t*‐tests. Pearson correlation coefficients and linear regression analyses were used to determine associations. Data are presented as means ± SEM. Statistical significance was defined as *P *<* *0.05.

## Results

### Body composition

**Figure 1:**
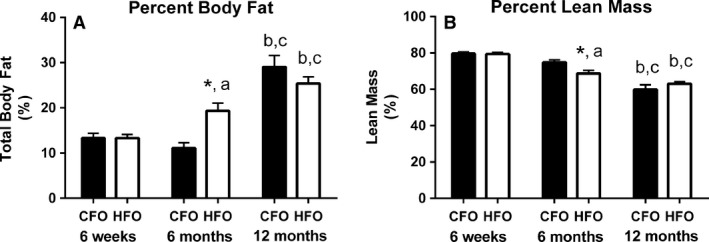
Body composition of CFO and HFO. Percent body fat (A) and percent lean mass (B) in CFO (black bars) and HFO (white bars) at 6 weeks, 6 months and 12 months of age. Six weeks (CFO:* n *=* *8, three females, five males; HFO:* n *=* *6, two females, four males), 6 months (CFO:* n *=* *6, three females, three males; HFO:* n *=* *6, three females, three males), 12 months (CFO:* n *=* *6, three females, three males; HFO:* n *=* *6, three females, three males). Offspring were randomized across age groups from CFO (six litters) and HFO (four litters). *significant difference between HFO and CFO at similar age (*P *<* *0.05). ^a^Significant difference between 6 months and 6 weeks for that paternal group (*P *<* *0.05). ^b^Significant difference between 12 months and 6 weeks for that paternal group (*P *<* *0.0001). ^c^Significant difference between 12 months and 6 months for that paternal group (*P *<* *0.05).

As expected, offspring total body weight increased with age (*P *<* *0.0001), regardless of paternal diet. There were no significant interactions between paternal group, age group, and biological sex for percent body fat or percent lean mass (*P *=* *0.48 and *P *=* *0.20, respectively); however, there were significant interactions between paternal diet and offspring age for percent body fat and percent lean mass (*P *<* *0.001, Fig. 1A,B). At 6 months of age, percent body fat was 74% higher (*P *<* *0.005, Fig. 1A), and percent lean mass was 8% lower (*P *<* *0.05, Fig. 1B) in the HFO compared to the CFO, despite offspring being on the same standard diet. No differences in fat or lean body composition were observed between HFO and CFO at 6 weeks or 12 months of age. Percent body fat increased 45% (*P *<* *0.05, Fig. 1A) between 6 weeks and 6 months of age, and then an additional 31% by 12 months of age (*P *<* *0.05, Fig. 1A) in the HFO mice. In CFO mice, percent body fat increased 162% from 6 months to 12 months (*P *<* *0.0001, Fig. 1A). Percent lean mass decreased 13% (*P *<* *0.0005, Fig. 1B) between 6 weeks and 6 months of age, and then decreased an additional 8% by 12 months of age (*P *<* *0.05, Fig. 1B) in the HFO mice. In CFO mice, percent lean mass decreased 20% from 6 months to 12 months (*P *<* *0.0001, Fig. 1B).

### Whole‐body insulin sensitivity

**Figure 2:**
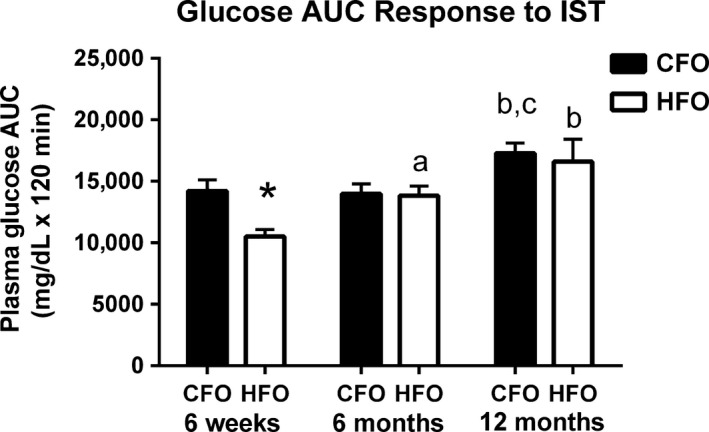
Insulin sensitivity in CFO and HFO. Insulin sensitivity was measured as the glucose AUC response to an insulin intraperitoneal injection. Measurements were done at 6 weeks, 6 months, and 12 months of age in CFO (black bars) and HFO (white bars). Six weeks (CFO:* n *=* *12, six females, six males; HFO:* n *=* *8, four females, four males), 6 months (CFO:* n *=* *8, four females, four males; HFO:* n *=* *8, four females, four males), or 12 months (CFO:* n *=* *6, three females, three males; HFO:* n *=* *6, three females, three males) of age in offspring. Offspring were randomized across age groups from CFO (six litters) and HFO (four litters). *significant difference between HFO and CFO at similar age (*P *<* *0.05). ^a^Significant difference between 6 months and 6 weeks for that paternal group (*P *<* *0.05). ^b^Significant difference between 12 months and 6 weeks for that paternal group (*P *<* *0.05). ^c^Significant difference between 12 months and 6 months for that paternal group (*P *<* *0.05).

There was no significant interaction between paternal group, age group, and biological sex for whole‐body insulin sensitivity (*P *=* *0.15); however, there was a significant interaction for whole‐body insulin sensitivity between paternal diet and offspring age (*P *<* *0.05). At 6 weeks of age, the glucose area under the curve (AUC) in response to insulin was 35% lower (*P *<* *0.05, Fig. 2) in HFO compared to CFO, despite the offspring being on the same standard chow diet. Glucose AUC did not differ between CFO and HFO at 6 months or 12 months (*P *>* *0.90). In the HFO, glucose AUC increased 31% (*P *<* *0.05) between 6 weeks and 6 months of age, and was 58% higher (*P *<* *0.001) at 12 months than at 6 weeks. In the CFO, glucose AUC was ~32% higher (*P *<* *0.05) at 12 months than at 6 weeks and 6 months.

### Skeletal muscle insulin signaling

#### Akt phosphorylation

**Figure 3:**
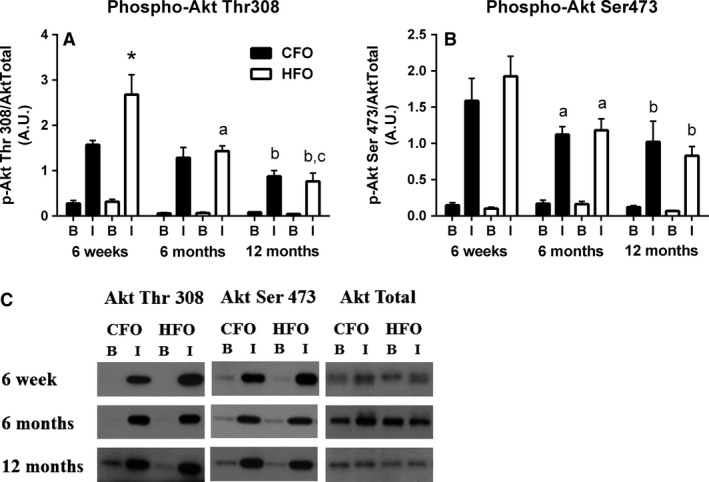
Skeletal muscle Akt phosphorylation in response to insulin in CFO and HFO. Soleus muscle Akt phosphorylation on sites threonine 308 (A) and serine 473 (B) in response to basal and insulin stimulation conditions in CFO (black bars) and HFO (white bars). Soleus AS160 protein representative blots for CFO and HFO at 6 weeks, 6 months, and 12 months (C). Six weeks (CFO:* n *=* *8, three females, five males; HFO:* n *=* *6, two females, four males), 6 months (CFO:* n *=* *6, three females, three males; HFO:* n *=* *6, three females, three males), 12 months (CFO:* n *=* *6, three females, three males; HFO:* n *=* *6, three females, three males). Offspring were randomized across age groups from CFO (six litters) and HFO (four litters). *Significant difference between HFO and CFOs at similar age and insulin condition (*P *<* *0.05). ^a^Significant difference between 6 months and 6 weeks for that paternal group (*P *<* *0.005). ^b^Significant difference between 12 months and 6 weeks for that paternal group (*P *<* *0.01). ^c^Significant difference between 12 months and 6 months for that paternal group (*P *<* *0.05).

There was a significant interaction between paternal group, age, and insulin condition (*P *<* *0.05); with post hoc analysis indicating that at 6 weeks of age, insulin‐stimulated Akt threonine 308 phosphorylation was 70% higher (*P *<* *0.05) in the HFO compared to the CFO (Fig. 3A). There were no other significant differences in Akt threonine 308 phosphorylation between HFO and CFO. Insulin‐stimulated Akt threonine 308 phosphorylation decreased 45% (*P *<* *0.005) between 6 weeks and 6 months of age, and a further 46% (*P *<* *0.05) between 6 and 12 months, in HFO (Fig. 3A). At 12 months of age, insulin‐stimulated Akt 308 serine phosphorylation was 71% and 44% lower (*P *<* *0.005) than that at 6 weeks, in HFO and CFO, respectively (Fig. 3A). Insulin‐induced phosphorylation of Akt on serine site 473 declined ~40% (*P *<* *0.001) between 6 weeks and 6 months of age, irrespective of paternal diet (Fig. 3B). At 12 months of age, insulin‐stimulated Akt 473 serine phosphorylation was ~47% lower than at 6 weeks of age (*P *<* *0.01), regardless of paternal diet (Fig. 3B). Basal Akt threonine 308 and serine 473 phosphorylation did not change with age and did not differ between CFO and HFO (Fig. 3). There were no age‐ or paternal‐related differences in total Akt protein in offspring.

#### AS160 phosphorylation

**Figure 4:**
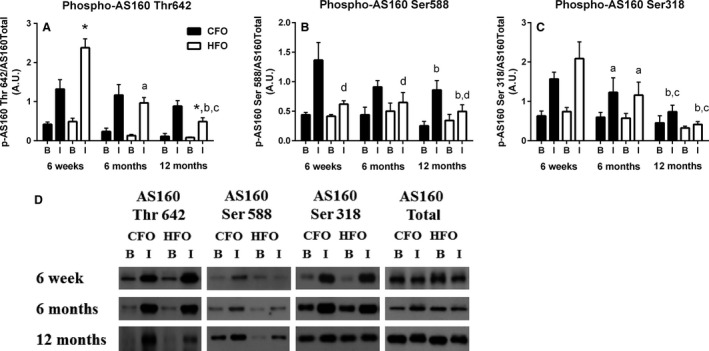
Skeletal muscle AS160 phosphorylation in response to insulin in CFO and HFO mice. Soleus muscle AS160 phosphorylation on sites threonine 642 (A), serine 588 (B), and serine 318 (C) in response to basal and insulin stimulation conditions in CFO (black bars) and HFO (white bars) mice. Soleus Akt protein representative blots for CFO and HFO at 6 weeks, 6 months, and 12 months (C). Six weeks (CFO:* n *=* *8, three females, five males; HFO:* n *=* *6, two females, four males), 6 months (CFO:* n *=* *6, three females, three males; HFO:* n *=* *6, three females, three males), 12 months (CFO:* n *=* *6, three females, three males; HFO:* n *=* *6, three females, three males). Offspring were randomized across age groups from CFO (six litters) and HFO (four litters). *Significant difference between HFO and CFOs at similar age and insulin condition (*P *<* *0.05).^a^Significant difference between 6 months and 6 weeks for that paternal group (*P *<* *0.05). ^b^Significant difference between 12 months and 6 weeks for that paternal group (*P *<* *0.05). ^c^Significant difference between 12 months and 6 months for that paternal group (*P *<* *0.05). ^d^Significant difference between CFO and HFO across age groups (*P *<* *0.005).

Examination of AS160 phosphorylation on site threonine 642 revealed a significant interaction between paternal group, age, and insulin condition (*P *<* *0.05). Insulin‐stimulated AS160 threonine 642 phosphorylation was 80% higher (*P *<* *0.05) in the HFO compared to the CFO at 6 weeks of age; however, at 12 months of age, phosphorylation was 45% lower (*P *<* *0.05) in the HFO compared to CFO (Fig. 4A). Insulin‐stimulated AS160 threonine 642 phosphorylation decreased by 59% (*P *<* *0.0005) between 6 weeks and 6 months, and a further 49% (*P *<* *0.05) at 12 months in the HFO (Fig. 4A). There were no significant changes in insulin‐stimulated AS160 642 threonine phosphorylation in CFO with age. With respect to AS160 serine 588 phosphorylation, there was a significant interaction between paternal group and insulin condition (*P *<* *0.001) as well as age and insulin condition (*P *<* *0.05). In HFO mice, the insulin‐induced phosphorylation of AS160 on serine site 588 was ~46% lower (*P *<* *0.005) than CFO at all age groups measured (Fig. 4B). At 12 months of age, the insulin‐stimulated AS160 serine 588 phosphorylation was ~35% lower (*P *<* *0.05) than at 6 weeks, regardless of paternal group (Fig. 4B). There was a significant interaction between age and insulin condition for AS160 serine 318 phosphorylation (*P *<* *0.01), with post hoc analysis demonstrating that insulin‐induced phosphorylation of AS160 on serine site 318 decreased ~36% (*P *<* *0.05) from 6 weeks to 6 months, and further decreased by ~52% (*P *<* *0.05) from 6 months to 12 months, irrespective of parental group (Fig. 4C). There were no age‐ or paternal‐related differences in total AS160 protein in offspring.

### Relationship between body fat and insulin signaling

**Figure 5:**
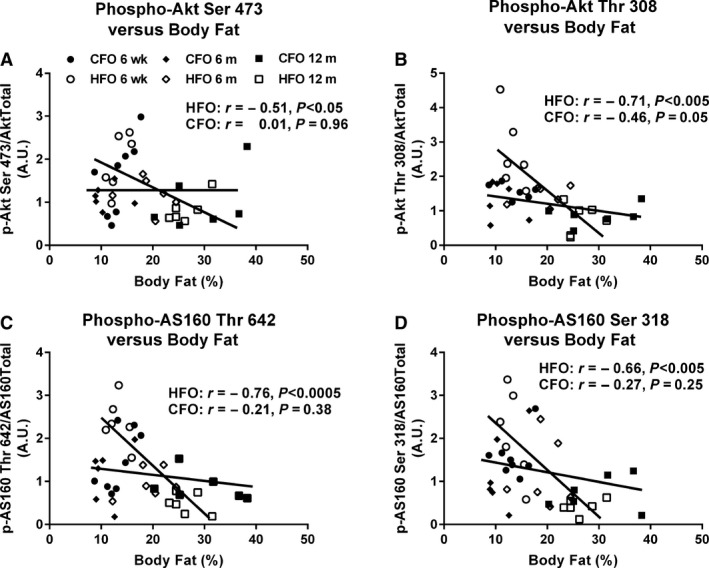
Relationship between skeletal muscle insulin‐stimulated signaling and body fat in CFO and HFO. Relationship between soleus insulin‐stimulated phosphorylation of Akt serine 473 (A), Akt threonine 308 (B), AS160 threonine 642 (C), AS160 serine 318 (D) and body fat in CFO (*n *=* *20, six litters, solid symbols) and HFO (*n *=* *18, four litters, clear symbols).

When all age groups were combined for the HFO, percent body fat was negatively associated with skeletal muscle insulin‐stimulated phosphorylation of Akt serine 473 (*r *=* *−0.51, *P *<* *0.05, Fig. 5A), Akt threonine 308 (*r *=* *−0.71, *P *<* *0.005, Fig. 5B), AS160 threonine 642 (*r *=* *−0.76, *P *<* *0.0005, Fig. 5C), and AS160 serine 318 (*r *=* *−0.66, *P *<* *0.005, Fig. 5D). There was a trend for body fat to be negatively related to insulin‐stimulated Akt phosphorylation on threonine 308 (*P *=* *0.05, Fig. 5B) in the CFO. Importantly, when controlling for biological sex, percent body fat in HFO remained strongly correlated with insulin‐stimulated Akt serine 473 (*r *=* *−0.55, *P *<* *0.05) and threonine 308 (*r *=* *−0.70, *P *<* *0.005), as well as, insulin‐stimulated AS160 threonine 642 phosphorylation (*r *=* *−0.75, *P *<* *0.001) and serine 318 (*r *=* *−0.67, *P *<* *0.005). Linear regression analysis determined that the slopes between CFO and HFO were significantly different for body fat and insulin‐stimulated phosphorylation of Akt threonine 308 (*P *<* *0.005), AS160 threonine 642 (*P *<* *0.005), and AS160 serine 318 (*P *<* *0.05).

## Discussion

In the current study, we demonstrate for the first time that the offspring of fathers fed a high‐fat diet (HFO) have increased insulin sensitivity (Fig. 2) and enhanced skeletal muscle insulin signaling (Figs. 3 and 4) early in life compared to the offspring from fathers fed a control diet (CFO). However, this health benefit is short‐lived, with the HFO becoming more susceptible to increases in body fat as they enter adulthood, despite being on a standard chow diet. Increases in body fat are related to reduced insulin signaling in the HFO, likely contributing to the abolishment of early insulin sensitivity and potentially increasing their risk for insulin resistance later in life.

A novel finding in the current study was that HFO had increased skeletal muscle insulin signaling (Akt, Fig. 3A and AS160, Fig. 4A) in response to an insulin challenge at 6 weeks of age. To our knowledge, this is the first study to examine the effects of paternal HFD on skeletal muscle insulin signaling. Given that skeletal muscle is responsible for approximately 70% of insulin‐stimulated glucose uptake (DeFronzo et al. 1981), it is likely the enhanced skeletal muscle insulin signaling (Figs. 3 and 4) contributed to the greater whole‐body insulin sensitivity observed at 6 weeks of age in the HFO (Fig. 2). Few studies have examined the effects of paternal HFD on whole‐body insulin sensitivity in offspring, and even fewer have investigated the effects prior to adulthood. However, a recent study by Lecomte et al. (2017) reported that 8‐week‐old offspring from paternal HFD‐fed rats had a tendency to have decreased fasting plasma insulin levels (~25% lower than control offspring), which normalized to controls by 6 months of age, supporting our findings that HFO exhibit increased insulin sensitivity early in life. Unfortunately, skeletal muscle was not examined in this previous study, but it was reported that circulating growth hormone (GH) levels were depressed in the young offspring from paternal HFD‐fed rats (Lecomte et al. 2017). We have previously demonstrated a link between GH levels and skeletal muscle IRS‐1 serine phosphorylation (Consitt et al. 2017), a marker of skeletal muscle insulin resistance. Although we do not expect our HFO to have had dramatically lower GH levels since we did not observe differences in total or lean mass, it is possible that even a minor decline could contribute, at least in part, to an enhancement in insulin action.

Researchers investigating the effects of maternal programming have determined that exposure to an early life stressor, including a HFD, increases the risk for metabolic disease, especially if later exposed to a secondary stress, known as the second‐hit phenomenon (Gallo et al. 2012; Cervantes‐Rodriguez et al. 2014; Fullston et al. 2015; Huypens et al. 2016). We propose that having a father on a HFD prior to conception contributes to the initial enhancement of skeletal muscle insulin signaling in the HFO, possibly as an attempt to protect against early onset insulin resistance if they are exposed to a HFD, similar to their father. In the current study, the offspring were fed a standard chow and *not* exposed to a nutritional stress, hence only exposed to the “single‐hit” of having their father exposed to a HFD. Therefore, it is conceivable that initial attempts to provide metabolic protection instead results in enhanced insulin sensitivity when not subjected to a similar dietary challenge as their father, at least early in life. It is evident that future research is necessary to investigate the mechanism(s) contributing to this enhanced insulin sensitivity in young HFO.

We have previously reported an inverse relationship between insulin‐induced skeletal muscle AS160 serine 588 phosphorylation and body fat percentage (Consitt et al. 2013); therefore, it was not completely unexpected that we observed lower insulin‐stimulated serine 588 phosphorylation on AS160 in the HFO at 6 months of age when they had increased body fat compared to CFO mice (Fig. 4B). However, it was surprising that this phosphorylation site was suppressed during insulin stimulation in HFO at 6 weeks, despite having increased insulin sensitivity, and no difference in total body fat between the two offspring groups at this age. Since HFO and CFO were treated and fed similarly throughout the study, we speculate that the differences observed with AS160 serine 588 phosphorylation at each age was the result of paternal dietary differences and possibly paternal body fat. Clearly, more research is needed to determine the mechanism and physiological significance of depressed AS160 serine 588 phosphorylation.

Despite the loss of early insulin sensitivity, HFO did not develop insulin resistance compared to the CFO, at least up until our final measurement (12 months of age). While a number of studies have reported the onset of whole‐body insulin resistance at 16–24 weeks of age in the offspring of paternal mice either overfed or fed a HFD (Pentinat et al. 2010; Fullston et al. 2013; Masuyama et al. 2016), others have reported no detrimental effects in offspring rats fed a standard chow between 8 weeks and 6 months (Ng et al. 2010; Lecomte et al. 2017), or at 15 weeks in offspring mice fed a HFD (Huypens et al. 2016). The reasoning behind these conflicting results remain unknown but may be related to differences in rodent species, rodent strain, postweaning diet, offspring sex, and/or age of measurements. Previous studies have suggested that maternal HFD may be more detrimental than paternal HFD on offspring glucose metabolism (Huypens et al. 2016; Masuyama et al. 2016). The results from the current study add to previous findings of normal glucose metabolism during early adulthood in offspring by demonstrating that HFO actually experience enhanced insulin sensitivity at an earlier age, which could contribute to the their lack of development of insulin resistance later in life. The mechanism(s) contributing to the loss of enhanced insulin sensitivity in HFO remain unclear, but may be related to their increased susceptibility to accumulate body fat earlier in life. It should also be noted that since we began to see some decrements in insulin‐stimulated AS160 phosphorylation in the soleus at 12 months of age (Fig. 4A), it is conceivable that negative whole‐body changes may be observed later in life.

Increased accumulation of body fat has long been associated with insulin resistance and impaired skeletal muscle insulin signaling (Goodyear et al. 1995; Jazet et al. 2008; Consitt et al. 2009). Our research supports the work of others (Fullston et al. 2013; Masuyama et al. 2016; Shi et al. 2017) documenting paternal metabolic programming as a potential cause for exaggerated gains in body fat during the transition into adulthood. Recently, Masuyama et al. (2016) reported that both male and female offspring fathered from mice fed a HFD had increased body fat compared to control offspring from 16 to 28 weeks of age. Similar to our findings, Fullston et al. (2013) reported that female offspring from paternal HFD mice had increased fat accumulation early in adulthood but was normalized to controls by 39 weeks of age. Given the detrimental effects of body fat and obesity on insulin sensitivity, it is not unreasonable to suggest that this premature accumulation of body fat at 6 months of age in our HFO contributed to the loss of earlier whole‐body and skeletal muscle insulin sensitivity at the same age. It remains unclear what physiological impact this earlier fat accumulation has on long‐term health, and whether our finding at 12 months of age that insulin‐stimulated AS160 phosphorylation on threonine site 642 was depressed was a consequence of premature fat gain. Our findings warrant future research to examine the effects of paternal high‐fat diet into the later stages of life to determine the metabolic consequences.

A novel finding in the current study was the relationship between increased body fat and impaired insulin signaling only in the skeletal muscle of HFO (Fig. 5), which was retained even after controlling for biological sex. This observation leads us to speculate that adipose location and intrinsic metabolic properties of adipose tissue, and not necessarily adipose total volume, contributed to decreased insulin signaling in HFO. Previous research has shown that the offspring of paternal high‐fat diet‐fed mice had increased body fat, as well as depressed circulating adiponectin and gene expression in adipose tissue at approximately 6 months of age, compared to controls (Masuyama et al. 2016). Further analysis revealed epigenetic changes to the adiponectin promoter region in the adipose tissue of the paternal HFD offspring. These findings are significant because adiponectin is known to enhance skeletal muscle glucose uptake (Yamauchi et al. 2002). Therefore, it is conceivable that the adipose tissue of our HFO synthesized and/or released less adiponectin levels, which contributed to the depressed insulin signaling. It has also been demonstrated that adipose tissue thermogenic capacity can be impaired by paternal hyperglycemia (Shi et al. 2017), signifying that adipose tissue phenotype can be influenced by paternal programming and could have contributed to our results.

A paucity of research exists regarding the role that offspring biological sex may have on metabolic outcomes of paternal high‐fat feeding. An early study suggested sex‐specific differences existed by reporting that paternal HFD‐induced glucose intolerance in female, but not male offspring rats, likely as a result of pancreatic beta‐cell dysfunction (Ng et al. 2010). Unfortunately, only female offspring data were published with the authors stating that initial pilot data demonstrated normal glucose tolerance in male offspring (S. F. Ng, unpublished) and acknowledged that it remained unknown if males exhibited similar defects in pancreatic gene expression as their female offspring counterparts. Others have determined that offspring from fathers fed a HFD develop glucose intolerance and insulin resistance, irrespective of offspring sex (Fullston et al. 2013; Wei et al. 2014). Inconsistencies have also been reported with offspring body fat in response to paternal HFD with female‐specific increases (Fullston et al. 2013) as well as increases (Masuyama et al. 2016) and no change (Huypens et al. 2016) in body fat, irrespective of offspring sex. A limitation of the current study was that our small sample size did not allow extensive analysis between male and female offspring and should be thoroughly investigated in future studies. That being said, our initial analysis provided evidence that changes in skeletal muscle signaling occurred independent of offspring sex (no significant biological sex interactions, and low estimated effect size). Unfortunately, no other studies have been conducted in skeletal muscle in response to paternal diet; however, both male and female offspring fathered by mice fed a low‐protein diet experienced similar increases in hepatic genes responsible for lipid and cholesterol biosynthesis (Carone et al. 2010). Taken together, it is evident that not all offspring peripheral tissue are susceptible to sex‐specific changes in response to paternal diets, and suggest highly metabolic prevalent peripheral tissue such as skeletal muscle and liver could experience changes irrespective of offspring sex.

## Conclusions

The current study demonstrates for the first time that HFO exhibit enhanced whole‐body insulin sensitivity and skeletal muscle insulin signaling early in life. Unfortunately, these benefits are short‐lived with an exaggerated gain in adipose tissue by 6 months of age. This study also highlights the detrimental effects of gaining fat if you are the offspring of a father that has consistently consumed a high‐fat diet prior to conception. The finding that HFO have increased insulin sensitivity early in life warrants future research to focus on identifying the cellular mechanisms that contribute to this so that these beneficial effects might be extended later in life. Taken together, this study provides novel and important insight into the role that paternal dietary choices may have on glucose and skeletal muscle metabolism in offspring.

## Disclosure statement

None to declare.
